# Igniting an autoinflammatory disease community: an interview with Ian Stedman

**DOI:** 10.1242/dmm.050642

**Published:** 2024-01-17

**Authors:** Ian Stedman

**Affiliations:** School of Public Policy and Administration, Faculty of Liberal Arts & Professional Studies, York University, 4700 Keele Street, Ross Building, S900, Toronto ON M3J 1P3, Canada

Systemic autoinflammatory diseases encompass a growing number of rare conditions, with prevalence ranging from 1 in 1000 to 1 in 1,000,000 people, depending on the specific disease, country, and population (Krainer et al., 2020). Autoinflammatory diseases are caused by overactivation of innate immune responses. This overdrive of pro-inflammatory responses results in varied symptoms, including fever, rash, arthritis and, in some cases, a serious build-up of protein in organs, which is associated with amyloidosis. These manifestations usually develop in childhood and, in many cases, delayed diagnosis and/or a lack of treatment options makes these conditions chronic, with a staggering impact on quality of life.

Ian Stedman is a strong advocate for people living with autoinflammatory diseases, having co-founded the Canadian Autoinflammatory Network (Réseau Auto-inflammatoire Canadien; Box 1) and acting as board chair for the Canadian Systemic Autoinflammatory Patient Advisory Group (Box 1) within Cassie+Friends, a Canadian charity for juvenile arthritis and other rheumatic diseases (Box 1). Ian and his daughter have a rare autoinflammatory disease called Muckle–Wells syndrome, which is one of the three types of Cryopyrin-Associated Periodic syndrome (CAPS). It is caused by inappropriate activation of the inflammasome, which functions as a hub for inflammatory responses within the cells, and increased secretion of the pro-inflammatory mediator interleukin-1 beta (IL1B). Following his diagnosis, Ian refocused his career in Law and is now an Assistant Professor of Canadian Public Law and Governance at York University, Canada, where he holds expertise in healthcare ethics and policy. He is also Vice Chair of the Canadian Institutes of Health Research, Institute of Genetics’ Advisory Board and a legal member on two Research Ethics Board panels at Toronto's Hospital for Sick Children (SickKids). It has recently been announced that Ian will be leading a team responsible for patient partnership, for the CIHR-funded Pan-Canadian Human Genome Library project. Here, he discusses his personal motivations to drive rare disease advocacy and emphasises the importance of broad cross-disciplinary approaches to improving healthcare and research.

**Box 1. Canadian Autoinflammatory Network and CAN-SAID**

The Canadian Autoinflammatory Network (Réseau Auto-inflammatoire Canadien) is a not-for-profit organisation based in Canada. Their core goal is to support people living with autoinflammatory diseases. The network is run by a team of passionate advocates, alongside a medical advisory board; they currently provide trusted information about rare autoinflammatory diseases for patients and families, including a symptom search tool that can lead people on the path to a diagnosis. The network was launched in 2022 and has plans to form partnerships with research and governance to enact patient-led research questions and to guide healthcare policy that serves all.

Cassie+Friends is a charity based in Canada that supports people living with juvenile arthritis and other rheumatic diseases. They have built a connected community across Canada and internationally, which provides support and in-depth education for patients and their families. They also support research by providing funding, and forming partnerships between patients and families, and researchers and clinicians. This research aims to meet patients' needs; it is varied in scope, including biomedical, mental health and socioeconomic research. As arthritis is a debilitating symptom for people with autoinflammatory diseases, Cassie+Friends established the Canadian Systemic Autoinflammatory Patient Advisory Group (Can-SAID). This group ensures that patients and parents affected by autoinflammatory diseases are fully incorporated into Cassie+Friends. Can-SAID have recently served as patient partners on an application for the Chan Zuckerburg ‘Rare As One’ grant.

**Figure:**
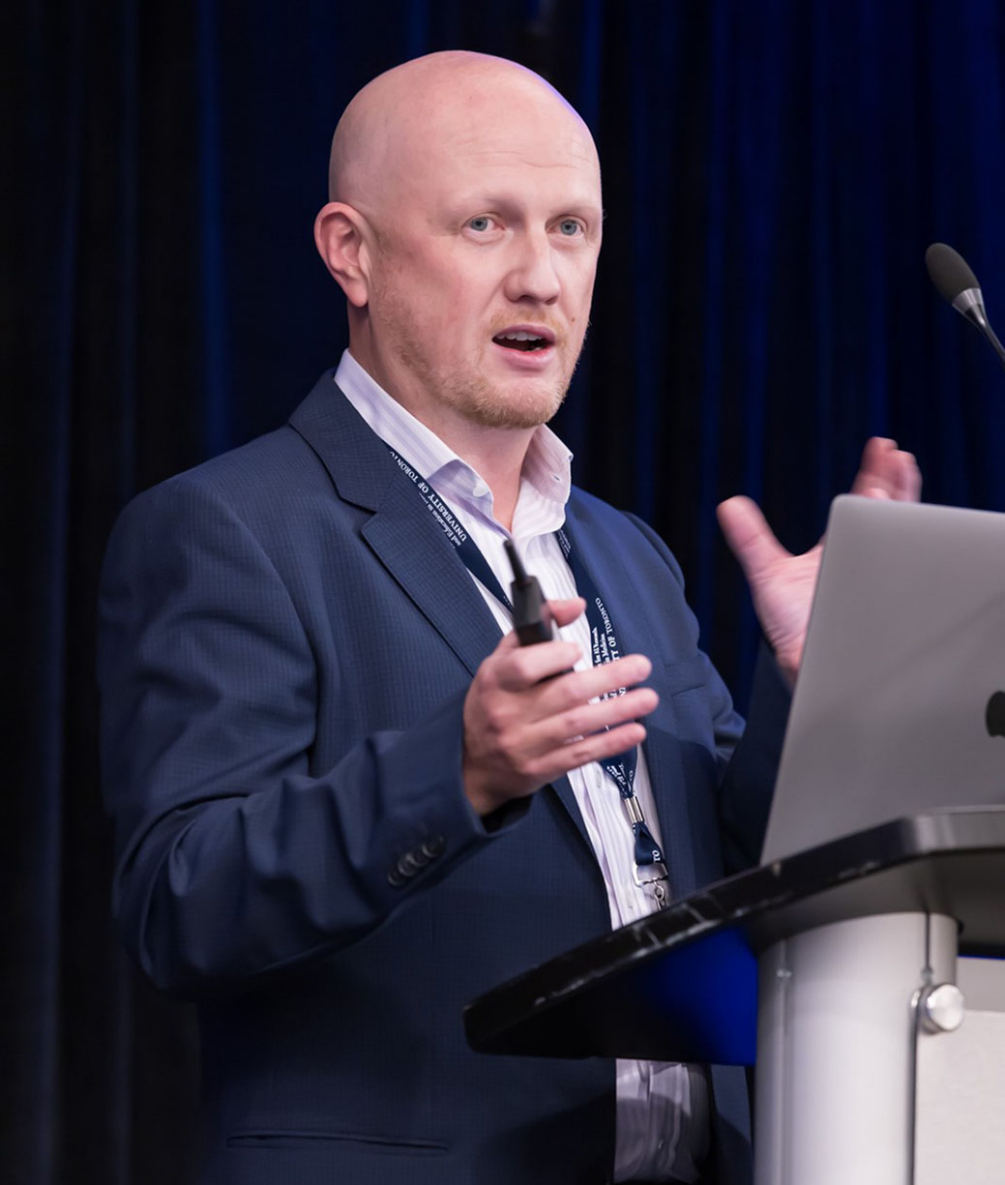
Ian Stedman

**Figure:**
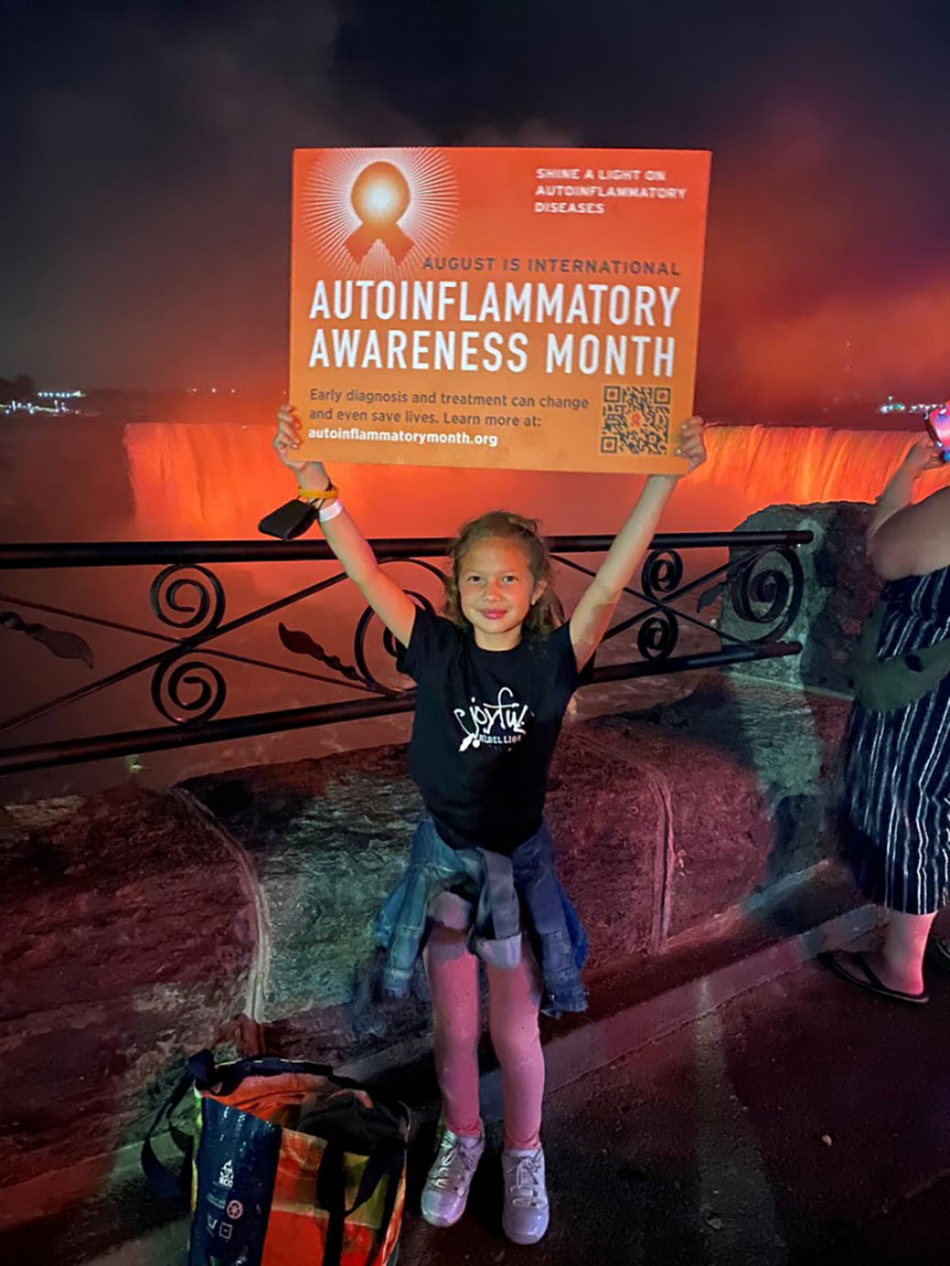
**Lia Stedman.** This image was provided by and with consent and assent from the Stedman family. Copyright 2024. All rights reserved. This image is not published under the CC-BY license of the article.

## How has your own experience with rare disease influenced your patient advocacy work and career?

When I was 32 years old, we had our first daughter and she presented with some symptoms that I had been experiencing my whole life – migraine headaches, cold sores, hearing loss, full body rashes, red eyes, periodic fevers and arthritis. She was born with the same rash as me and, although she had started walking at nine or ten months, within that first year she stopped walking and started crawling again. She couldn't articulate what was wrong, but I could tell it was due to pain in her joints. For 32 years, I had been bouncing around between doctors looking for a diagnosis, but never got real answers. When I went to university, I'd given up trying to find answers and just found ways to manage the symptoms as they happened. Seeing my daughter's symptoms woke me up. I started doing research in evenings and weekends, staying up until two o'clock every night reading everything I possibly could, to try to find out what might be wrong with my daughter. But I don't have a background in healthcare or medicine, so this stuff was way beyond me. When we eventually got the diagnosis, it was because I was searching through pictures of people's skin conditions online and trying to figure out what looked like mine. I emailed Dr Ron Laxer at SickKids [Toronto] explaining our story, with the pictures of the rashes, and he was able to confirm the diagnosis of Muckle–Wells syndrome through genetic sequencing when I was 32 and my daughter was one.

Before our daughter was born, I was already a lawyer and had moved into the public sector – to the Office of the Integrity Commissioner of Ontario – after practicing law in a small firm. I'm sure it doesn't surprise anyone that, if you're living with a chronic illness, finding a job that's a little less stressful than private practice really bodes well for you. In the public sector, I had better access to benefits and flexibility in my schedule, and people were a little more understanding of my needs. After our diagnosis, I started trying to find time in my life to learn more about healthcare and understand more about the disease we had been diagnosed with. It was during this transition period – when I was working full time and spending another 40 h a week learning about healthcare, rare diseases, health policy and advocacy (and never sleeping!) that everything changed. We sat down as a family and said, “Okay, we probably need to refocus my career, because this isn't sustainable”. I thought it would be really nice if I could do something within my career that aligned more closely with what I felt was necessary for our family in terms of healthcare advocacy and policy work. I quit my job and went back to school to get a Master's degree and then a PhD in law from York University's Osgoode Hall Law School, North York, Canada. I'm now a Professor in the School of Public Policy and Administration at York University, and a lot of my work is about healthcare, medical technologies and rare diseases. The work I do is inspired by my experiences, and what I've learned about myself and the healthcare system along the way. So, the diagnosis has completely turned my life on its head – for the better – and I now have more of a sense of purpose.

## What motivated you to co-found the Canadian Autoinflammatory Network and how does this organisation plan to influence research in autoinflammatory diseases?

It took me a long time to start a not-for-profit. There was a lot of pressure to do it, but for those of us who are patients and got their diagnosis later in life – maybe with families and full-time jobs – to then take on advocacy work is not a small task. Doing it in an organised and structured manner, like building an organisation, is a tonne of work.

One of the goals of the Canadian Autoinflammatory Network is for researchers who are interested in autoinflammatory diseases to know where to go to talk to patients about their research questions and to disseminate their research. Our aim is that we establish partnerships with the medical and research communities, so that relevant research proposals don't advance without a partnership with patient advocacy groups from the beginning. Also, we shouldn't have these conversations post proposal or post funding but before the funding proposal goes in. I want researchers to sit down with patients and ask if their research will help them. Our goal is to create a visible space where we can offer that kind of empowerment for the researchers so that their work has impact.

I think the real value will be in making sure that, when those researchers do publish, they have a network to disseminate that research. Historically, individual rheumatologists will have a few patients they're trying to diagnose. We're getting to a point where we can create professional networks with the Canadian Autoinflammatory Network and Cassie+Friends, to help disseminate the research to different clinicians, who are busy but need to stay connected. That's something we can do to help facilitate growth within research.

I think there's going to be greater power in the collective voice.

## Rare disease patient advocacy groups tend to encompass broader disease groups in order to have a stronger influence, but should the research itself be more focused on individual disorders, like Muckle–Wells syndrome?

When my daughter and I were diagnosed, I wanted to figure out how to advocate for the Muckle–Wells syndrome community. I realised that it wouldn't be good enough just to advocate for Muckle–Wells, because these autoinflammatory diseases have so many similarities regarding their symptoms. There are a lot of people who present as if having an autoinflammatory disease but, when their genomes are sequenced, they can't get a genetic diagnosis. These people should have a place where they can meet other people who have had a similar experience, and who can help them navigate the system and give advice on what treatments and care have worked for them. The goal of the Canadian Autoinflammatory Network is to create an advocacy space for everyone diagnosed with or suspected of having an autoinflammatory disease in Canada. I think there's going to be greater power in the collective voice.

I think one of the challenges right now is that they've described so many autoinflammatory disorders, many of which have only been diagnosed in one or two patients. So, there are a lot of autoinflammatory diseases that don't have enough patients to run a clinical study. But if you can better understand one of them – like Muckle–Wells syndrome – then you're probably going to better understand the underlying mechanisms of all of these diseases.

If we're calling something ‘rare’, governments feel more justified in dismissing it. As science advances, I think we're going to end up moving away from the language of rare disease. What if, instead, we say that every person's needs are unique?

## How can rare disease research help inform mechanisms and treatments for more common disease?

We've seen some recent examples of this, like the speculation that Ilaris^®^ – the drug used to treat Muckle–Wells syndrome by blocking IL1B – is potentially beneficial for people with COVID who have severe lung inflammation. But there was not enough incentive to study that further because the drug is so expensive – $16,000 per injection – so it just didn't seem feasible for the pharma companies to pursue it. Also, if you give someone with gout Ilaris^®^, their symptoms subside quite significantly – but gout can be treated in significantly cheaper ways. Nevertheless, in doing the basic research, we're learning a lot about those common disease pathways. I think as we see the tiny variations in how the pathways cause symptoms in different diseases, then we will open ourselves to understanding inflammation better and how certain proteins implicated in these diseases work.

One of the things that is starting to have a huge effect on funding is that diseases we thought of as common – like cancers – are being subdivided into things that are rare. We thought breast cancer was just breast cancer, but now we know, through genetics, that it's not one size fits all. It's stories like that, that are changing the funding model. Also, if we keep calling a disease rare, we're disincentivizing research, if we look at it from the view of an economic model. If we're calling something ‘rare’, governments feel more justified in dismissing it. As science advances, I think we're going to end up moving away from the language of rare disease. What if, instead, we say that every person's needs are unique? I think the future of healthcare is in personalization. Every person who walks into healthcare has to be treated like a zebra, nobody like a horse – every single person has to be treated as a unique individual. Otherwise, the healthcare system won't work for everyone and people will continue to fall through the cracks.

How do we build this system? Maybe we can work together to advance data collection, data sharing and data analytics, and to create pathways through healthcare that are more inclusive, yet efficient and less burdensome on our wallets. Funders and governments will want to find some efficiencies, especially with an ageing population that is demanding so much more of our healthcare system.

[…] ethics is an important part of making sure that we move forward without excluding and further marginalising the vulnerable.

## With rapid advances in technology, research and medicine, should collaboration between biomedical researchers and experts in law and ethics become more common?

Absolutely. One of the challenges we have in Canada with our healthcare system is that we signed this Constitution in 1867, and it says that healthcare delivery and service is going to be provided provincially − with privacy also being a provincial matter. Therefore, data collection and data privacy policies are provincial, and data are not readily shared nationwide. What we've learned from rare diseases is that, unless there is a widely used registry, we're not collectively gaining information from the progression of a patient's disease and their diagnosis and treatment. This is because our healthcare systems don't communicate. Therefore, the importance of having ethicists, lawyers, policy experts and social scientists sharing the table with STEM scientists is so we can come up with some really cool ideas. A geneticist can sequence all the genomes they want but we're never going to be able to properly learn from these genomes in Canada, because of this constitutional barrier. The policy and law professionals can look for solutions, i.e. to find ways around these archaic constitutional structures. This can bridge gaps and build more-inclusive healthcare systems and research environments comprising provincial and territorial synergy. This is exemplified by the recently announced Pan-Canadian Human Genome Library.

Then, in terms of ethics, if we're going to sequence everyone's genomes, we better have had conversations about what to do with the findings. For example, if – after having sequenced my mom's genome with her permission – finding out that she and I are susceptible to a disease, what should be done with that information? I didn't consent to sequencing my genome but the conclusion is that I might be at risk too. For those reasons, I think ethicists are incredibly important. There's also a history of research on vulnerable people, when it should not have been done or, at least, should not have been done the way it had been done. So, ethics is an important part of making sure that we move forward without excluding and further marginalising the vulnerable. If we're going to sequence a million genomes, we should probably have some sense of the diversity of the genomes that we're sequencing, so that, when we have this massive collection of data, it isn't just to benefit people who look like me – white and of European descent.

So, yes, lawyers, ethicists and policy professionals, need to be a part of the conversation. But the challenge is that the language used in these professions is different to that of scientists. As a lawyer, I spent a decade of my life learning about the law but now I must learn about science so I can positively and correctly contribute to discussions about how to advance science and create new systems. That doesn't happen without inclusive collaborative approaches in research and academia.

## What recent advances in the rare disease space have you found most interesting?

When I was doing my PhD, I was working with a colleague named Michael Brudno, who was doing work using artificial intelligence and genomics. Dr Brudno started a company called PhenoTips that uses advanced algorithms to analyse genomic data to help locate the gene or genes that might be responsible for a person's symptoms – i.e. their phenotype. Ultimately, he was using machine learning to help diagnose people. At one point, he asked if the company could take my story about my symptoms as I'd described them online and run it through their algorithm to see how long it would take the PhenoTips system to diagnose me. I got my handwritten medical records from all the doctors I'd ever seen – something like 88 separate pages and 300 entries – and PhenoTips digitised it, ran it through the software and had a possible diagnosis for me by doctor visit number eight. Why do we expect doctors to know all 9000 rare diseases, when we can create these algorithms to help diagnosis? That was a eureka moment for me about five years ago. Data analytics may have tremendous potential to change how we get to diagnosis.

I think patient engagement is good, but patient partnership is more important. Patients ought to be included before research happens.

## What are patients most concerned about when it comes to research in rare autoinflammatory diseases?

Most people with autoinflammatory diseases spend their lives navigating their symptoms, which doesn't leave much time for flourishing in life or living the life they've always imagined. For the most part, people with a chronic illness just want it to go away or want it to be managed in a way that isn't burdensome. My daughter and I have access to the medicine developed by Novartis called Ilaris^®^ that is delivered by subcutaneous injection every eight weeks. When we started being treated with Ilaris^®^ all our symptoms went away overnight. As far as I'm concerned, it's a miracle drug. But my anxiety about having symptoms didn't go away for months, I would wake up in the middle of the night, having nightmares about being covered in hives. I would get up in the morning and the first thing I would do was – like I had done for 30 years – stand in front of the mirror saying “Are my eyes red? Is my skin red? Do I have to take some painkillers for my foot if I have another arthritic flare?”. So, despite the symptoms disappearing overnight, the psychosocial effects had not gone away immediately. I think our community is getting more interested in how research could help us physically but also psychosocially. I'm hopeful for a future where more energy is put into understanding that part of the burden and running a healthcare system that is attentive to it.

If you’d ask me, “We're going to work on Muckle–Wells syndrome but can only work on two symptoms at a time, so which two burden you the most?”, you might expect that it's my hearing loss of 20% in one ear, or the fact that I have joint swelling that limits my mobility. However, I would tell you it's not those symptoms that have the greatest physical impact, it's the ones that have the greatest psychosocial impact − like my eyes being constantly bloodshot, and my skin rashes. If you were to eliminate those, I could actually live a much more full and flourishing life, without constantly, in my own head, thinking about my identity being wrapped up with my symptomology. That's why patients should be included from the get-go! If having access to a patient group like ours, answers to those questions can be obtained in anonymous surveys. Organising research around patient partnership and creating a patient advocacy space, allows us to create opportunities for both the researchers, and the community of patients and families to get involved in research. I think patient engagement is good, but patient partnership is more important. Patients ought to be included before research happens.

When I was searching for a diagnosis, I was chasing the symptoms that had the biggest impact on my psychosocial existence. So, I spent 30 years going to every single dermatologist and ophthalmologist who would see me for my skin and for my eyes. Then I found out in my 30 s that, even though skin presentation was the most common symptom in these diseases, 80% of people with autoinflammatory diseases are diagnosed by a rheumatologist because they all have joint pain/arthritis. If trying to enable people to access to care quicker, one has to understand how patients proceed through the system, a journey that is informed by those symptoms that actually matter to them individually.

If I'm being a little more granular, it is necessary to look at what it means to take a medication like Ilaris^®^. For me, it means that I have to put a needle in my daughter. Personally, I'm okay with getting my medicine administered by a needle if it takes away all the pain I've experienced for 30 years. But my daughter never really understood that. To her, the needle represents something about her that's different. It's a burden for her because she doesn't have the comparison I had, which was 32 years of pain and discomfort. So, for her, the needle is a big negative part of her life. It would be nice if there was an oral medicine or – I know we're a long way from this – if genome editing was an option for treatment. I'm hoping that science gets us there in a safe and effective way because it'd be really nice for my daughter to be able to travel the world and not worry about how she's going to store her medicine and how to have access to it. She could just go off, live her life and flourish, and not have her identity and sense of self so deeply tied to her diagnosis.

## Conclusions

The collective voice for rare disease advocacy holds immense power and can drive research, funding and healthcare policy in the right direction. With the wide array of symptoms associated with autoinflammatory diseases, it is important for researchers and clinicians to understand those that have the greatest impact on patients' lives. This dictates how patients interact with healthcare systems, as it can impact their diagnosis and their desired research priorities. The Canadian Autoinflammatory Network holds a space for partnerships between patients and researchers, and urges a cross-disciplinary approach to tackling the core challenges in rare disease. By integrating law, ethics and governance with research and healthcare, we can begin to understand the true burden of rare disease and develop creative solutions. This would also allow us to navigate a research and healthcare landscape that, increasingly, relies upon genomics and enables us to approach this in the most ethical and equitable way.

Ian also emphasises the importance of personalisation in healthcare to benefit people with rare diseases but also those with increasingly subdivided common diseases. Fundamental research into the genotype and phenotype of rare and common diseases is essential to understand the shared and divergent mechanisms of disease between these patients, which would inform their personalised treatment and care.

Disease Models & Mechanisms (DMM) thanks Dr Ian Stedman for his willingness to be interviewed, and for sharing his unique experiences and perspectives with us. Ian was interviewed by Kirsty Hooper, Features & Reviews Editor for DMM, and this interview has been edited and condensed with the interviewee's approval.
